# Multilayer Density Analysis of Cellulose Thin Films

**DOI:** 10.3389/fchem.2019.00251

**Published:** 2019-04-16

**Authors:** Carina Sampl, Katrin Niegelhell, David Reishofer, Roland Resel, Stefan Spirk, Ulrich Hirn

**Affiliations:** ^1^Institute for Paper, Pulp and Fibre Technology, Graz University of Technology, Graz, Austria; ^2^CD-Laboratory for Fibre Swelling and Paper Performance, Graz University of Technology, Graz, Austria; ^3^Institute of Solid State Physics, Graz University of Technology, Graz, Austria

**Keywords:** multilayer analysis, surface plasmon resonance, atomic force microscopy, cellulose thin film, X-ray reflectivity

## Abstract

An approach for the multilayer density analysis of polysaccharide thin films at the example of cellulose is presented. In detail, a model was developed for the evaluation of the density in different layers across the thickness direction of the film. The cellulose thin film was split into a so called “roughness layer” present at the surface and a “bulk layer” attached to the substrate surface. For this approach, a combination of multi-parameter surface plasmon resonance spectroscopy (SPR) and atomic force microscopy (AFM) was employed to detect changes in the properties, such as cellulose content and density, thickness and refractive index, of the surface near layer and the bulk layer. The surface region of the films featured a much lower density than the bulk. Further, these results correlate to X-ray reflectivity studies, indicating a similar layered structure with reduced density at the surface near regions. The proposed method provides an approach to analyse density variations in thin films which can be used to study material properties and swelling behavior in different layers of the films. Limitations and challenges of the multilayer model evaluation method of cellulose thin films were discussed. This particularly involves the selection of the starting values for iteration of the layer thickness of the top layer, which was overcome by incorporation of AFM data in this study.

## Introduction

The past decades have seen tremendous efforts to explore and to understand processes occurring at surfaces of materials, which was accompanied by the development of new surface sensitive techniques and methodologies. These techniques are either capable of determining the surface properties such as morphology, topography, chemical structure, and composition, or they are utilized to monitor dynamic phenomena taking place at interfaces, such as adsorption, modification, or wetting, to name a few (Vickerman and Gilmore, 2009; Zaera, 2011).

Atomic force microscopy (AFM) (Binnig et al., 1986), for instance, is an imaging technique for the investigation of surfaces on an atomic scale by scanning the surface using a cantilever with a sharp tip while it is maintained at a constant force or a constant height above the sample. Nowadays, the observation of surfaces in real-time using fast scanning devices (one frame per second and faster) allows for visualizing dynamic biological processes at interfaces. For example, enzymatic degradation of biopolymers such as cellulose can be monitored and videos of the degradation process can be acquired (Giessibl, 2003; Jalili and Laxminarayana, 2004; Niegelhell et al., 2016).

Surface plasmon resonance (SPR) spectroscopy is a surface sensitive technique that is able to monitor processes in real-time as well. In contrast to atomic force microscopy, it detects changes in the chemical environment, i.e., the refractive index *n*, near a metal surface (~100 nm) (Raether, 1977). The technique is based on the resonance of surface plasmons, which originates from the oscillation of charge densities on the metal surface caused by freely moving electron gas (Ritchie, 1957). Acquisition of SPR spectra is accomplished by focusing a *p*-polarized light source onto a metal surface (e.g., a glass slide coated with a thin gold layer) and recording the intensity of the reflected light by a detection system (e.g., photodiode array detectors). State of the art multi-parameter SPR devices, acquire spectra in dependence of the incidence angle of the light, which allows for the investigation of processes in real-time and in varying ambient media (i.e., gas or liquid) at different wavelengths (i.e., 670 and 785 nm) in a single experiment. The resulting curves are evaluated by a multilayer fitting procedure, whereby thickness (*d*) and refractive index (*n*) of the examined layers are derived (Geddes et al., 1994). In a standard SPR spectrometer, where the measurement is performed with only a single wavelength, determination of both -layer thickness and refractive index- is not possible without assuming or knowing one of the values. Multi-parameter SPR (MP-SPR) systems measuring at two or more wavelengths overcome this problem (Peterlinz and Georgiadis, 1996; Liang et al., 2010).

When observing phenomena with SPR spectroscopy in real-time, the change in refractive index related to the whole sample is detected. In most cases, this information is sufficient because it allows for monitoring adsorption processes at the interfaces, in terms of deposited mass and adsorption kinetics. Many examples do exist which investigate biomolecule deposition on cellulose thin films mainly with the aim to establish sensor systems or antifouling surfaces (Orelma et al., 2011, 2012; Niegelhell et al., 2016, 2017, 2018; Strasser et al., 2016; Mohan et al., 2017; Weißl et al., 2018). Since water incorporation into the film changes its refractive index, swelling has been investigated by SPR as well for the whole film (Kontturi et al., 2013). However, when it comes to more complex questions, i.e., whether the swelling of such a thin film is different on the “surface” than in the “bulk,” other approaches with more complex fitting procedures are required. Up to now, the fitting procedures used in SPR data evaluation have only been used to describe the cellulose film as a single layer. When splitting the film into more than one layer, more information is required for the fitting procedure.

Herein, the investigation of such a multilayer analysis approach to evaluate density variations of different layers in thin films, at the example of cellulose, is presented. A model describing the cellulose thin film as a multilayer system was developed. The implementation of AFM data into the multilayer fitting model was of outmost importance in order to obtain starting values for the fitting procedure concerning thickness of bottom and top layer. This combination of analysis methods and the presented multilayer density analysis approach yielded spatial resolution along the z-axis of the examined films. Compositions of the entire films as well as differences between the surface regions and the bulk were evaluated. The approach was compared to X-ray reflectivity (XRR) measurements of cellulose thin films, which were also analyzed to obtain thickness and density data for a multilayer cellulose film system.

## Theoretical Background

### Determination of Thickness and Refractive Index of Cellulose Thin Films: The 2-Wavelength Method (2-λ-Method)

The simulation of a sample measured at a single wavelength provides a refractive index (*n*)—thickness (*d*) continuum (*n* decreases when *d* increases) without a unique solution, since the surface plasmon wave vector *k*_*sp*_ is dependent on both entities.

(1) $$\begin{array}{r} k_{SP}\;\propto \;n\;*d \end{array}$$

In order to determine a unique solution, measurements at two wavelengths resulting in two different sets of *n - d* continua are required. Since the refractive index is dependent on the wavelength, the *n - d* continuum measured at one wavelength can be shifted to the other wavelength (Peterlinz and Georgiadis, 1996). The unique solution is found at the intersection of these continuum solutions.

(2) $$\begin{array}{r} k_{SP1}=n_{\text{λ}1}*d\;and \end{array}$$

(3) $$\begin{array}{r} k_{SP2}=n_{\text{λ}2}\;*d \end{array}$$

where

(4) $$\begin{array}{r} n_{\text{λ}2}=\left(n_{\text{λ}1}+\;\frac{dn}{d\text{λ}}\;\left({\text{λ}}_{2}-{\text{λ}}_{1}\right)\right)and \end{array}$$

(5) $$\begin{array}{r} k_{SP1}=n_{\text{λ}1}*d\;and \end{array}$$

(6) $$\begin{array}{rcl} \;k_{SP2} & = & \left(n_{\text{λ}1}+\;\frac{dn}{d\text{λ}}\;\left({\text{λ}}_{2}-{\text{λ}}_{1}\right)\right)\;*d \end{array}$$

The dependence of refractive index on the wavelength, i.e., chromatic dispersion (*dn/d*λ), is approximated to be linear for relatively small wavelength changes (a few hundred nm).

For cellulose, the *dn/d*λ at 670 and 785 nm is −0.0338 and −0.0204 μm^−1^, respectively (Kasarova et al., 2007). The average, −0.0271 μm^−1^, was used in the calculations as a chromatic dispersion value of pure cellulose. The corresponding value for air is −0.00000856 μm^−1^ (Ciddor, 1996). The *n - d* curves of the cellulose thin films obtained by the two wavelengths were plotted in the same graph. The *n - d* curve of the measurement performed at 785 nm was shifted by the *dn/d*λ value of cellulose and by the *dn/d*λ value of the ambient medium (air). The intersection points of the shifted curves (air and cellulose) measured at 785 nm with the curve measured at 670 nm results in two unique solutions. An average (*n*_*film*_) of the *n* values obtained by the intersection points was determined. The proportion of cellulose of the volume of the film, *a* corresponding to the obtained *n*_*film*_ value, was calculated according to Equation (7):

(7) $$\begin{array}{r} n_{film}=\;{a\cdot n}_{cellulose}+\;{\left(1-a\right)\;\cdot \;n}_{medium} \end{array}$$

where *n*_*cellulose*_ is 1.466 (Kasarova et al., 2007) and *n*_*medium*_ is 1.00028 for air (Ciddor, 1996).

Based on a, the value of *dn/d*λ was corrected by Equation (8) (Kontturi et al., 2013):

(8) $$\begin{array}{r} dn/d{\text{λ}}_{film}=\;{a\cdot dn/d\text{λ}}_{cellulose}+\left(1-a\right)\;\cdot \;dn/d{\text{λ}}_{medium} \end{array}$$

Then, the intersection point of the *n - d* curve measured at 670 nm and the *n - d* curve shifted from 785 to 670 nm with the new *dn/d*λ results in the unique solution (*n* and *d*) of the cellulose thin film (for a detailed example see **Figure 4** in section results and discussion). The composition *a* of the film was determined by Equation (7). The densities of the films were calculated from the composition and the density values of pure cellulose and air (see Supplementary Material) similar to Equation (7).

### Multilayer Analysis of Cellulose Thin Films

**Figure 1:**
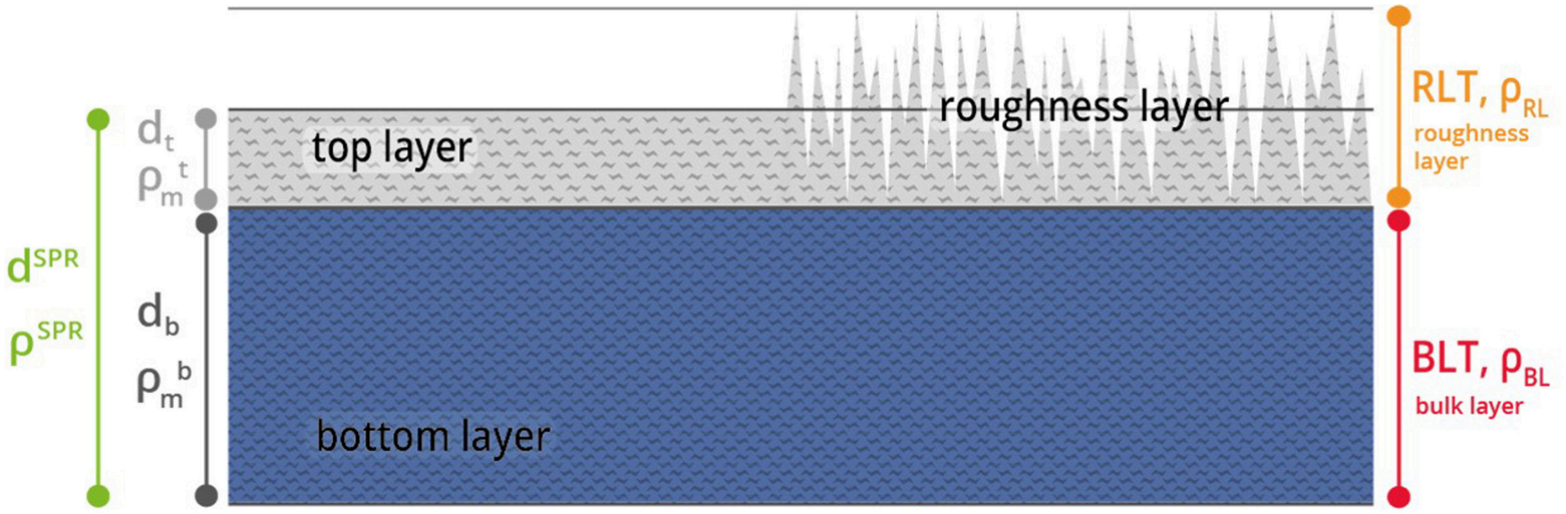
Graphic description of the multilayer analysis model. The film consist of a roughness layer *RL* (gray) and a bulk layer *BL* (blue) with certain thickness *d* and density ρ. The thickness *d*^*SPR*^ and density ρ^*SPR*^ of the entire film are obtained via MP-SPR spectroscopy. The film is then split into a top layer (*d*_*t*_, $\rho_{m}^{t}$) and a bottom layer (*BL, d*_*b*_, $\rho_{m}^{b}$).

The procedure to obtain thickness *d* and the density (via refractive index *n*) of a thin film has been described in detail in the last section (Theoretical Background). These MP-SPR measurements are the basis for the multilayer analysis of thin film samples (Figure 1). The investigated film is split into two layers, namely the roughness layer (*RL*) and the bulk layer (*BL*). Thickness and density are determined for each layer.

The roughness layer *RL* describes the layer that contains the boundary between the film and the ambient medium i.e., the roughness of the film. The *RL* is composed of cellulose and air, the material fraction (*mf*) refers to the percentage of material within the volume of the *RL*.

**Figure 2:**
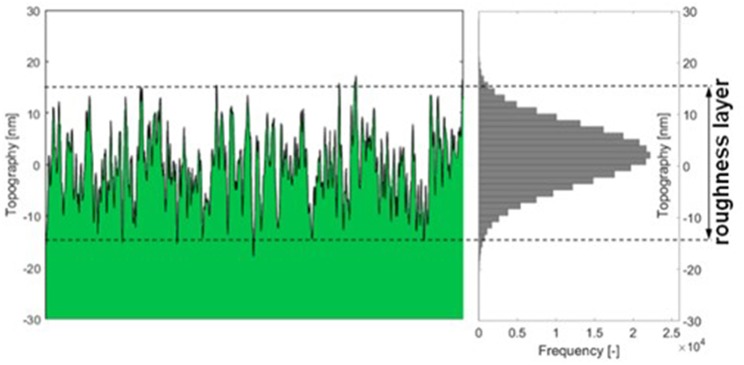
Graphic description of AFM data evaluation: the thickness of the roughness layer (*RLT*) comprises 95% of the topography range measured with the AFM. The material fraction ${mf}_{RL}^{AFM}$ is the percentage of material (green) within the roughness layer.

Thin film data required to make use of the model is attained by MP-SPR spectroscopy. This data does not contain information on surface roughness, i.e., the roughness layer *RL* is treated as a flat layer with thickness *d*_*t*_ and density $\rho_{m}^{t}$. The roughness layer thickness (*RLT*^*AFM*^) as well as its material fraction (${mf}_{RL}^{AFM}$) were acquired from AFM topography experiments. AFM data was evaluated as illustrated in Figure 2 and implemented into simulations. A detailed description of the AFM evaluations is shown in the experimental section and in the Supplementary Material.

### Multilayer Data Analysis for 2-Layer Thin Films Analysis

**Figure 3:**
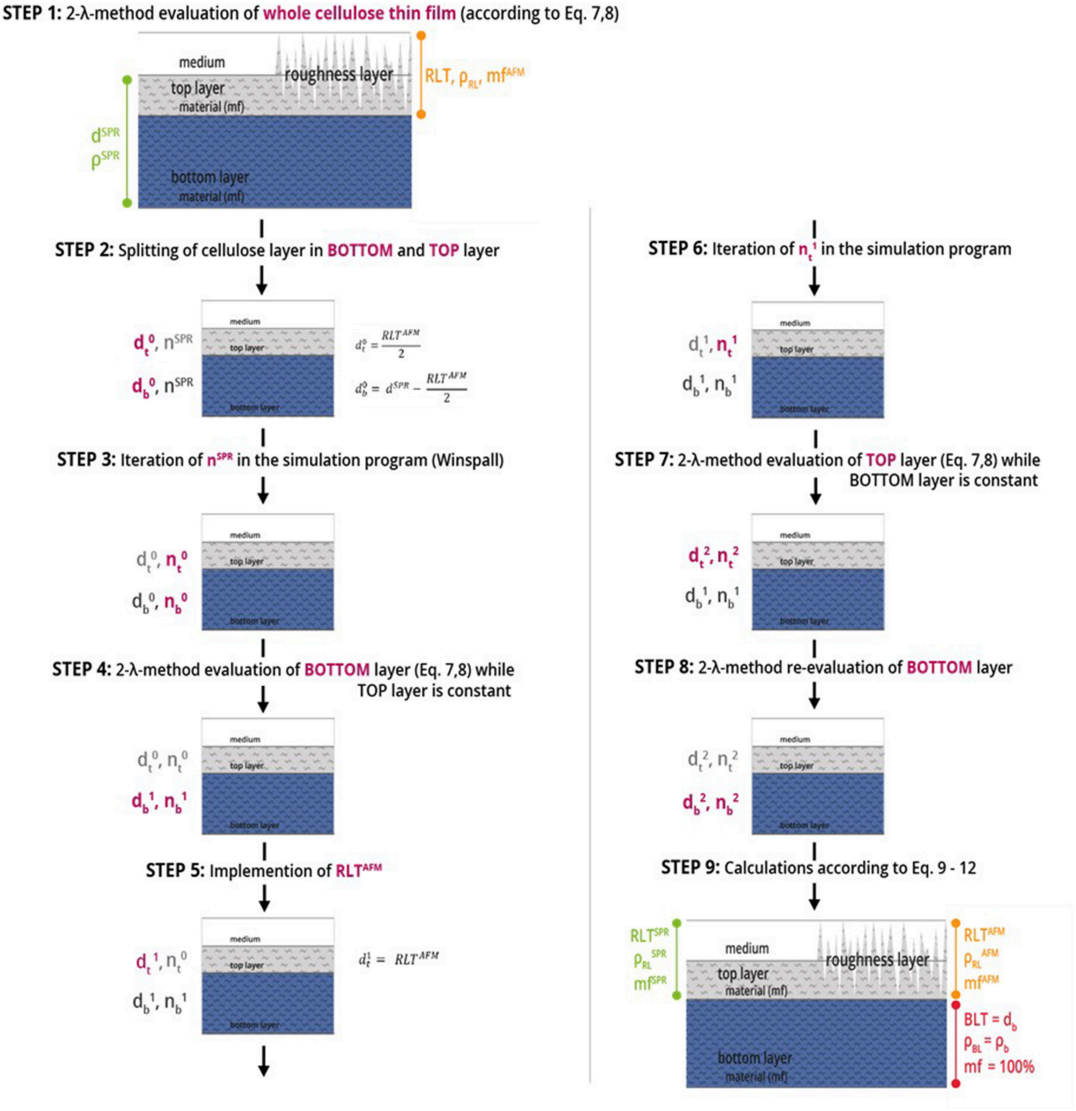
Steps of multilayer analysis of cellulose thin films performed by means of MP-SPR spectroscopy, where *d* refers to the thickness [nm], *n* is the refractive index, *RL* is the roughness layer, *RLT* is the roughness layer thickness, *mf* is the material fraction [%], *BL* is the bulk layer, *BLT* is the bulk layer thickness and ρ is the density [g·cm^−3^]. The indices *t, b* (subscript), *AFM* and *SPR* (superscript) stand for top layer and bottom layer. The number of iteration is given in the superscript, e.g., for thickness *d* as *d*^0^*, d*^1^*, and d*^2^. Red text refers to the values changed in the respective evaluation step.

We are using the modeling software Winspall to analyze film thickness and refractive index (density) from SPR measurements. The key approach of performing multilayer analysis is modeling the thickness for two layers of material instead of one layer of material in Winspall, in combination with the 2-λ method for determining *n* and *d* of the respective layers. Starting from a 1-layer analysis and using the AFM measurement results as starting values for the roughness layer thickness as starting values for the iteration a sequence of models is built to successively refine and confirm the results for refractive index (density) and film thickness in top- and bottom layer of the thin film. A step-by step description for this modeling procedure is shown in Figure 3. Finally, equations 9 to 12 are used to calculate the final results.

(9) $$\begin{array}{r} \rho_{RL}^{AFM}=\frac{mf^{AFM\;}\;\left[\%\right]}{100}\;\cdot \;\rho_{m}^{t} \end{array}$$

(10) $$\begin{array}{r} RTL^{SPR}=\frac{d_{t}\;\cdot \;100}{cellulose\;content\;\left[\%\right]} \end{array}$$

(11) $$\begin{array}{r} \rho_{RL}^{SPR}=\;\frac{mf^{SPR}\;\left[\%\right]}{100}\;\cdot \;\rho_{m\;}^{t} \end{array}$$

(12) $$\begin{array}{r} mf^{SPR}=\frac{d_{t\;}\cdot \;100}{RLT^{AFM}}\;. \end{array}$$

Here $\rho_{RL}^{AFM/SPR}$ is the density of the roughness layer and *mf*^*AFM*/*SPR*^ is the material fraction calculated from AFM or MP-SPR data. RLT is the roughness layer thickness, *d*_*t*_ is the thickness of the top layer and $\rho_{m}^{t}$ is the density of the top layer from MP-SPR multilayer evaluation.

The nine steps of the 2-layer analysis as shown in Figure 3 are described below. Notation is explained in the caption of Figure 3. For a detailed example over all 9 steps of the analysis of a cellulose thin film, see Table S1.

**STEP 1:** Starting point is a 1-layer analysis of the entire film according to the 2-λ method. The result is thickness *d*^*SPR*^ and density ρ^*SPR*^ (refractive index *n*^*SPR*^, respectively) for the entire cellulose film.

**STEP 2:** The film is split into two layers –the top and bottom layer- denoted as $d_{t}^{0}$ and ${\text{d}}_{b}^{0}$. Whereas, the starting $d_{t}^{0}$ values for evaluation are derived from AFM data with $d_{t}^{0}$ = RLT^AFM^/2. Starting $d_{b}^{0}$ value is set to *d*^*SPR*^—$d_{t}^{0}$. The refractive index of the entire cellulose thin film *n*^*SPR*^ is chosen as starting refractive index value for both, top ($n_{t}^{0}$) and bottom layer ($n_{b}^{0}$).

**STEP 3:** Iteration for the corresponding refractive indices; Result: refractive indices $n_{t}^{0}$ and $n_{b}^{0}$.

**STEP 4:** Evaluation of the bottom layer by 2-λ method yielding $d_{b}^{1}$ and $n_{b}^{1}$, while keeping the values for the top layer ($d_{t}^{0}$, $n_{t}^{0}$) constant.

**STEP 5:** The roughness layer thickness obtained from AFM imaging (*RLT*^*AFM*^) is implemented into the model as new thickness value for the top layer ($d_{t}^{1}$).

**STEP 6 and 7:** The corresponding refractive index ($n_{t}^{1}$) is iterated in the simulation program and $d_{t}^{2}$ and $n_{t}^{2}$ of the top layer is evaluated by the 2-λ method, while the bottom layer values ($d_{b}^{1}$, $n_{b}^{1}$) are kept constant.

**STEP 8:** Evaluation of the bottom layer by the 2-λ method while keeping the new values for the top layer ($d_{t}^{2}$, $n_{t}^{2}$) constant, in order to validate the simulations.

**STEP 9:** To calculate the roughness layer's thickness (*RLT*), the density ($\rho_{RL}^{SPR/AFM}$) and the material fraction (*mf*^*SPR*/*AFM*^) data from AFM and SPR as iterated above are calculated using Equations (9–12).

## Experimental

### Materials

Trimethylsilyl cellulose (TMSC, DS_Si_ = 2.8, Avicel, M_w_ = 185.000 g·mol^−1^, M_n_ = 30.400 g·mol^−1^, PDI = 6.1 determined by SEC in chloroform) was purchased from TITK (Rudolfstadt, Germany). Chloroform (CHCl_3_, 99.3%), hydrochloric acid (HCl, 37 %), hydrogen peroxide (H_2_O_2_, 30% in water) and sulfuric acid (H_2_SO_4_, 95%) were purchased from Sigma Aldrich and used as received. MilliQ water (resistivity = 18 MΩ·cm) from a Millipore water purification system (Millipore, USA) was used for all experiments. A total of three films was measured with SPR and AFM and the 2-layer analysis was performed.

### Substrate Cleaning and Film Preparation

SPR sensor slides—glass sensors with chromium adhesion layer (~5 nm) and gold coating (~50 nm) (CEN102Au)—were obtained from Cenibra, (Bramsche, Germany). In order to remove adventitious carbon, SPR sensors were cleaned before use by treatment with piranha solution (freshly prepared from sulfuric acid and hydrogen peroxide in a 3:1 (v/v) ratio) over a period of 10 min. Afterwards the SPR slides were extensively rinsed with MilliQ water and dried in a stream of nitrogen. The silicon wafer substrates (native oxide layer, 1.4 × 1.4 cm^2^) for the XRR measurements were cleaned with “piranha” acid [H_2_SO_4_:H_2_O_2_ = 7:3 (v/v)] for 30 min and neutralized afterwards with distilled water.

Cellulose thin films were prepared by spin coating trimethylsilyl cellulose (0.75 wt% in CHCl_3_) onto the gold slides. One hundred microliters of TMSC-solution were deposited on the substrate and then rotated for 60 s at a spinning speed of 4,000 rpm and an acceleration of 2,500 rpm·s^−1^. TMSC was converted to cellulose by treatment with hydrochloric acid (HCl) vapor. The TMSC films were placed into a petri dish (diameter 5 cm) containing 3 ml of 10% HCl. The dish was covered with its cap and the films were exposed to the HCl vapors for 15 min. The regeneration was verified by water contact angle and ATR-IR measurements as reported elsewhere (Kontturi et al., 2003; Woods et al., 2011; Mohan et al., 2012a,b).

### Multi-Parameter Surface Plasmon Resonance (MP-SPR) Spectroscopy

Two-wavelength MP-SPR spectroscopy experiments were performed with an MP-SPR Navi^TM^ 210A Vasa instrument (by BioNavis Ltd., Tampere, Finland) equipped with two light source pairs providing 670 and 785 nm in each of the two measurement channels. All measurements were performed using a full angular scan (39−78°, scan speed: 8°·s^−1^) in three parallels. SPR data was processed with BioNavis Dataviewer software. The full angular scans were simulated with the optical fitting software Winspall 3.01 (which is freely available from the Max-Planck Institute for Polymer Research (Mainz, Germany), http://www2.mpip-mainz.mpg.de/groups/knoll/software, 12.6.2013). The multilayer fitting approach is based on the Fresnel equations and the recursion formalism. The SPR signal of the pure sensor surface was simulated first in order to obtain the background for the subsequent evaluation of the cellulose thin film. The two wavelengths cross point analyses were performed both using Microsoft Office Excel 2010 and Origin 8.6.

### Atomic Force Microscopy (AFM)

AFM imaging was performed in atomic force microscopy tapping mode with a Veeco multimode scanning probe microscope (Bruker, USA). The images were scanned using silicon cantilevers (NCH-VS1-W, Nanoworld, Switzerland) with a resonance frequency of 320 kHz and a force constant of 42 N·m^−1^. All images were processed using Nanoscope software package (V7.30r1sr3, Veeco).

The roughness layer thickness RLT^AFM^ is evaluated from the topography images measured by AFM, compare Figure 2. The roughness layer is the region of the cellulose film where both, material and ambient medium (water or air) are found, i.e., the region containing the boundary between cellulose film and surrounding. The thickness of this region depends on the roughness of the film. We have defined the roughness layer as the z-directional layer containing 95% of the surface roughness. For calculation, the histogram of the topography distribution is evaluated. From each edge of the histogram, 2.5% of the topography values are clipped off, the topography range comprising the remaining 95% of height values is defined as the roughness layer thickness. We are not using 100% of the values because the topography distribution is close to normally distributed which means there are always some far outliers which would bloat the roughness layer beyond the true surface interaction range.

### X-Ray Reflectivity XRR

X-ray reflectivity measurements were performed using a PANalytical Empyrean goniometer system with radiation produced by a copper sealed tube (λ = 0.154178 nm). The primary side of the reflectometer was equipped with a 20 mm beam mask, a multilayer mirror, a 1/32° slit, and an automatic beam attenuator. On the secondary side, a receiving slit of 0.1 mm and a Soller slit of 0.02 rad were used in front the PANalytical PIXEL3D point detector. The sample stage was a domed DHS 900 from Anton Paar (Resel et al., 2003), equipped with a SHT15 humidity sensor to monitor the relative humidity and the temperature during the measurements. The relative humidity (RH) was controlled using a S-503 humidity generator from Michell instruments. For each humidity step an equilibration time of 30 min was accomplished. XRR measurements were performed in the 2θ region 0.030–9.999° with a step size of 0.006°. The evaluation of the data was performed with the X'Pert Reflectivity (Panalytical, C_6_H_10_O_5_ for cellulose was used) software package providing information on the electron density, layer thickness, and the roughness of the films by applying (Parratt, 1954) formalism and the disturbance term of Nevot–Croce(Nevot and Croce, 1980). A three layer model was required to fit the experimental data of the cellulose film (50% relative humidity, same as in laboratory where the SPR spectrometer is located), resulting in a very thin layer at the Si-cellulose interface (*d* = 0.6 nm, ρ = 0.8 g cm^−3^), a bulk layer (*d* = 43.9 nm, ρ = 1.39 g cm^−3^) and a surface layer (*d* = 4.2 nm, ρ = 1.09 g cm^−3^). Total film thickness was 48.7 nm, with a density of 1.41 g cm^−3^ at 50% relative humidity. For the sake of comparison, data obtained at other humidity levels (0, 25, and 70% was acquired. The difference in layer thickness compared to the gold substrate stems from the different wettability of the silicon wafer (which is required for XRR) leading to higher thicknesses.

## Results and Discussion

### Characterization and Preparation of Cellulose Thin Films

**Figure 4:**
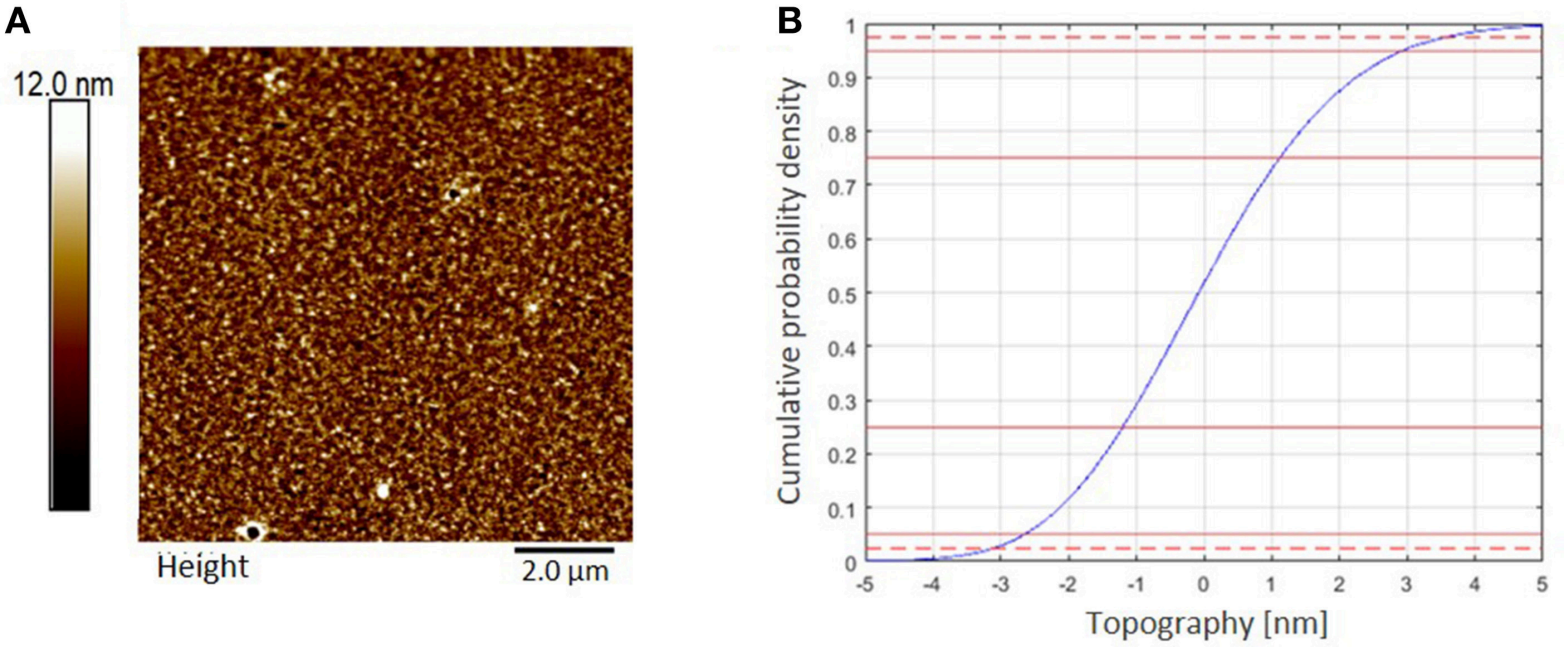
**(A)** AFM topography image (10 × 10 μm^2^) of a cellulose thin film spin coated from CHCl_3_ measured in air at ambient conditions, z-scale 30 nm. RMS roughness (*R*_*q*_) is 1.7 nm. **(B)** AFM topography evaluation. Roughness layer thickness *RLT*^*AFM*^ = 6.4 nm, 95% of the topography range are indicated by the dashed red lines in the cumulative topography distribution.

Cellulose thin films were prepared by spin coating of trimethylsilyl cellulose (TMSC), dissolved in chloroform (CHCl_3_), and subsequent conversion to cellulose by a regeneration step completed with HCl vapors, cleaving off the silyl-groups by formation of TMSCl (Kontturi et al., 2003; Kontturi and Lankinen, 2010). The completed conversion to cellulose was proven by ATR-IR spectroscopy, proving the disappearance of bands related to the TMS group (υ_SiC_, δ_SiOC_ at 1,250 and 852 cm^−1^) while the typical OH stretching vibrations (υ_OH_) for cellulose at 3,200–3,600 cm^−1^ appeared (Ehmann et al., 2015). The AFM images display smooth, homogeneous surface topography (Figure 4). Evaluation of thickness from AFM-imaging results in a *RLT* (roughness layer thickness) of 6.4 nm and a cellulose material fraction (*mf*^*AFM*^) of 47.9 % in that film.

### 2-Layer Analysis of Thickness and Density in Thin Films

**Figure 5:**
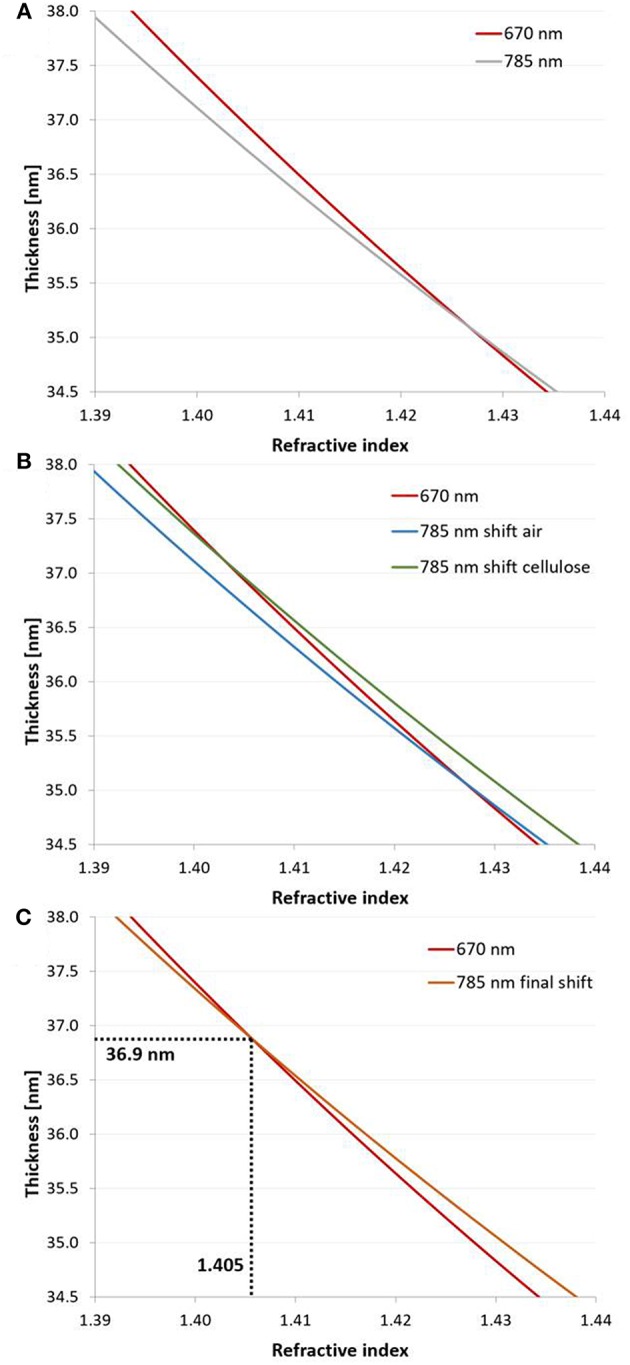
2-λ method: **(A)** Continuous solutions (*n* – *d* curves) obtained at 670 and 785 nm at the example of a dry cellulose thin film (film 1 in Table S3) spin coated from CHCl_3_. **(B)** The curve measured at 785 nm was shifted by the *dn/d*λ of air (−0.00000856 μm^−1^) and cellulose (−0.0271 μm^−1^) in order to determine the composition of the film. The new *dn/d*λ value for the film consisting of air and cellulose was re-estimated from the composition (average of refractive indices at both intersection points) with equations 7–8. **(C)** The *n* – *d* curve recorded at 785 nm was shifted by the resulting *dn/d*λ to obtain the unique solution (*d* = 36.9 nm, *n* = 1.406, *a* = 89 % cellulose) for the investigated thin film.

Thickness and refractive indices of three individual cellulose thin films were first determined for the entire film using the 2-λ method (Figure 5) as described above. In principle, cellulose thin films can be prepared with high reproducibility and only slight variations in film thickness. For example, the thickness evaluation of three thin films applying the 2-λ method led to just minor deviations (*d*^*SPR*^ of 36.4 ± 0.5 nm). The refractive indices at 785 and 670 nm are 1.408 ± 0.028 and 1.410 ± 0.024, respectively, which can be used to determine the cellulose content *a* in the whole film (Equation 7, 88.8 ± 0.1%). Based on that result, the film was subjected to multilayer density analysis as outlined in the previous sections (Figure 3). The first result was that the refractive indices of the top and bottom layer did not yield the same result. We found much smaller values for the top layer (*n*_*t*_ = 1.237 ± 0.034 at 670 nm; 1.242 ± 0.030 at 785 nm) than for the bottom layer (*n*_*b*_ = 1.421 ± 0.012 for 670 and 1.420 ± 0.011 for 785 nm). As a consequence, the cellulose content in the bottom layer ($a_{b}^{SPR}$ = 91.8 ± 0.1 %) is much larger than in the top layer ($a_{t}^{SPR}$ = 52.2 ± 0.1 %). The thickness of the entire films and the sum of the top and bottom layer thicknesses obtained by our multilayer analysis are consistent (one layer analysis: *d*^*SPR*^ = 36.4 ± 0.5 vs. two layer analysis 36.5 ± 0.6 nm (bottom *d*_*b*_ = 33.7 ± 0.7 and top layer *d*_*t*_ = 2.8 ± 0.2 nm). Further, the density of the whole film (ρ^*SPR*^ = 1.34 ± 0.09 g·cm^−3^) is very well-represented by the individual densities of the top and bottom layers (bottom $\rho_{m}^{b}$ = 1.39 ± 0.04 g·cm^−3^ and top $\rho_{m}^{t}$ = 0.76 ± 0.10 g·cm^−3^). The results from the multilayer density analysis using data obtained from AFM and MP-SPR spectroscopy experiments are depicted in Table 1. Evaluation results of the three cellulose thins films are shown in Table S2. Further, each individual thin film is evaluated in a multiple manner and compared to the other films, in order to demonstrate stability of the fitting approach and indicate deviations (see Table S3).

**Table 1:** Comparison of results determined by MP-SPR spectroscopy (see Table S3), AFM and XRR of cellulose thin films measured in air (average from three films).

	**AFM**	**MP-SPR**	**XRR**
d_b_[nm]	–	33.7 ± 0.7	43.9 ± 0.3
$\rho_{\text{m}}^{\text{b}}$ [g·cm^−3^]	–	1.39 ± 0.04	1.39 ± 0.10
d_t_ [nm]	–	2.8 ± 0.2	4.2 ± 0.1
$\rho_{\text{m}}^{\text{t}}$ [g·cm^−3^]	–	0.76 ± 0.10	1.05 ± 0.10
RLT [nm]	6.4 ± 0.2	5.5 ± 1.4	–
ρ_RL_ [g·cm^−3^]	0.37 ± 0.06	0.33 ± 0.07	–
mf [%]	48 ± 2	43 ± 7	–

*RLT^SPR^, mf^SPR^ and $\rho_{\text{RL}}^{\text{SPR}/\text{AFM}}$ were calculated according to Equations (9-−12). Note that for XRR, films featured a different thickness due to the use of a different substrate and the values that have been given in the table were obtained at 50% relative humidity*.

### Comparison of AFM, SPR, and XRR Data

The resulting data from AFM, MP-SPR spectroscopy, and XRR was compared to validate the employed strategy for multilayer analysis. The material fraction (*mf*, composed of cellulose and air) and *RLT* were acquired by AFM. The cellulose content, i.e., the density of the material ($\rho_{m}^{t}$), and the thickness of the layer consisting of material, i.e., the top layer (*d*_*t*_), were obtained from MP-SPR spectroscopy. For each technique, values which correspond to the same parameter determined by the other technique ($\rho_{RL}^{AFM}$, $\rho_{RL}^{SPR}$, *RLT*^*SPR*^, and *mf*^*SPR*^) were calculated from the measured results (*mf*^*AFM*^, *RLT*^*AFM*^, *d*_*t*_ and $\rho_{m}^{t}$), according to Equations (9–12). The outcome of these calculations is summarized in Table 1. While there are some minor deviations, the results are very consistent for AFM and 2-layer MP-SPR approach. The deviations can to some extent be attributed to the different measurement areas of the applied techniques and small variations in the cellulose thin films. For instance, AFM covers an area of 10 × 10 μm^2^, whereas the area of the MP-SPR spectroscopy lasers (Ø = 0.6 mm) is ~0.28 mm^2^.

**Figure 6:**
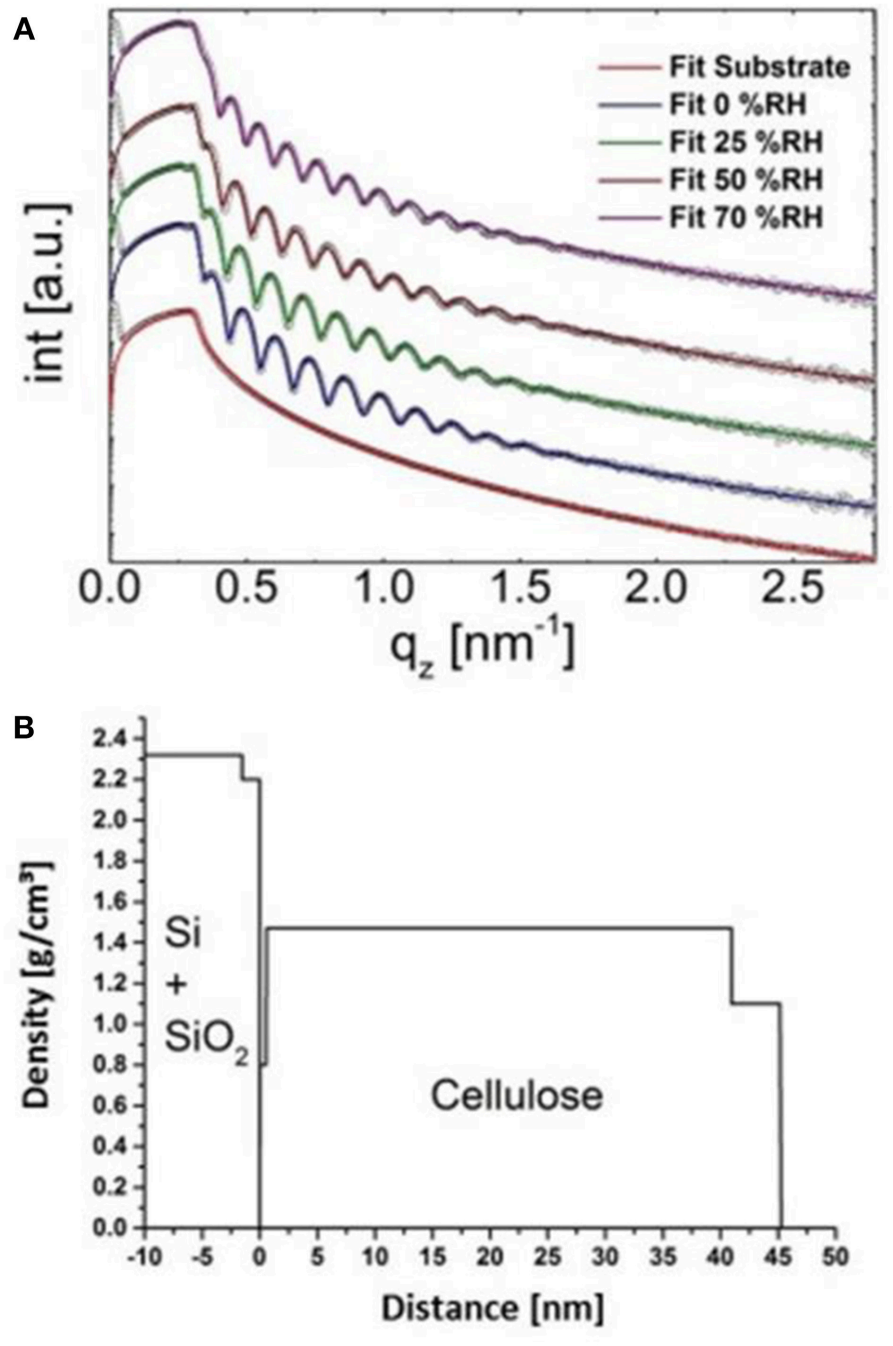
Fits of the XRR curves of the cellulose thin film at different humidity levels **(A)** and concomitant density profile through the layer (at 0% humidity, **B**).

X-Ray reflectivity was employed to further validate the approach. However, the results are not directly comparable in terms of absolute layer thicknesses since gold substrates (used in SPR spectroscopy) cannot be used for that technique. Instead, films have been deposited on silicon wafers which have a different surface free energy, leading to different cellulose film thickness. Further, the film thickness is a function of humidity level; by increasing humidity levels, layer thickness increases (from 0 to 70% r.h. thickness increase by 14%, see Table S4, for full data and Figure 6A for the XRR curves including fitting). At the same humidity levels as in the labs where the AFM and the SPR spectrometer are located (50% r.h.), we observed a surface layer with reduced density (thickness: 4.2 nm, ρ: 1.05 g· cm^−3^) as well as a bulk layer with a higher density (thickness: 43.9 nm, ρ: 1.39 g cm^−3^). The share of the top layer on the overall layer thickness is 7.7 and 9.1% for cellulose thin films on gold and silicon wafers, respectively. Given the different experimental setup and the difference in the substrates, the XRR data is in excellent agreement with the multilayer density approach using AFM/SPR. The densities of the prepared films are in the range of molecular dynamics simulations on amorphous cellulose (1.34–1.39 g cm^−3^) (Mazeau and Heux, 2003; Chen et al., 2004) but significantly lower than those of reported bulk amorphous substrates (1.48 g cm^−3^) (Mark, 1982). It should be noted that XRR revealed a thin layer (0.6 nm) at the cellulose-Si interface with rather low density (0.8 g cm^−3^). This is a known phenomenon for many polymer films but could not be revealed by SPR spectroscopy. The density profile of the layered system obtained by XRR is depicted in Figure 6B.

In summary both, MP-SPR and XRR yield a similar layer structure of the cellulose films, with a lower material density in a few nanometers thick top layer of the film and a higher density in the bulk of the film.

### Potential and Limitations of the Method

Finally, the limitations and requirements of the multilayer density analysis approach need to be elucidated. The crucial point in the presented multilayer approach is to have appropriate starting values for the thickness of the bottom and the top layer (*d*_*t*_^0^ and *d*_*b*_^0^) for the data analysis in order to obtain physically meaningful results. Unique solutions for given *RLT/2* values were only obtained when the starting values for the thickness were in between *d*^*SPR*^ and the value obtained by iteration of the simulation program ($d_{t}^{0}$). A randomly chosen starting value for the *RLT* does not lead to a result corresponding to the real *RLT* of the thin film. Furthermore, some randomly chosen starting values of the *RLT* did not even yield intersection points of the *n - d* curves. However, by using AFM data providing starting values for *RLT*, physically meaningful results can be obtained, which was confirmed by XRR. The importance of implementing AFM data as starting values for the fitting process is shown in more detail in Table S1. Comparability of fitting results and deviations within multiple evaluations of the individual thin films is described in Table S3 (Supplementary Material). The main advantage of SPR is that it can be used with a wide range of liquids while by XRR this is a rather challenging task. The multilayer analysis of thin films to explore swelling processes in different liquids is a potentially powerful application and will be applied in future reports.

## Conclusion

We demonstrated a multilayer density analysis of thin films at the example of cellulose in air. We showed that the combination of AFM and SPR data allows for analyzing the density variation and thickness in surface near regions of cellulose thin films. The key finding, a layer with reduced density and a thickness of a few nanometers at the top of the cellulose film, was confirmed by XRR measurements on similar films. We believe that this approach has the potential to reveal the density in multilayer structures of a wide range of materials (biopolymer films, porous thin films), e.g., for density analysis in thin film structures or for water uptake during swelling of cellulose with different types of liquids. Still, the approach features some limitations and further improvements need to be accomplished. A major shortcoming is that the starting values used for the splitting of the layer are very important to get meaningful results in the fitting procedure, i.e., an educated guess is required, which was provided by AFM in this study. A further limitation is the used modeling software which seems to have minor problems with numerical stability (see Tables S2, S3), when inappropriate starting values were used. Further improvements of the approach in future studies will focus on more robustness toward the starting values used for the fitting procedures. A promising approach is to employ 4- or 6- different wavelengths in MP-SPR measurements and to use this additional information together with adequate modeling software for an improved multilayer analysis of thin films.

## Author Contributions

All authors listed have made a substantial, direct and intellectual contribution to the work, and approved it for publication.

### Conflict of Interest Statement

The authors declare that the research was conducted in the absence of any commercial or financial relationships that could be construed as a potential conflict of interest.

## Supplementary Material

Supplementary Material Supporting information is available including AFM topography evaluation, background MP-SPR curves,additional simulation experiments for multilayer analysis, results of multilayer analysis and comparison of multiple evaluations.
