# Supplementary material for: Multilayer Density Analysis of Cellulose Thin Films
Source: Front Chem. 2019 Apr 16;7:251. doi: 10.3389/fchem.2019.00251 (PMC6476991; doi:10.3389/fchem.2019.00251)
Supplement: Supplementary Material — Supporting information is available including AFM topography evaluation, background MP-SPR curves,additional simulation experiments for multilayer analysis, results of multilayer analysis and comparison of multiple evaluations. [file Table_1.docx]

*SUPPLEMENTARY MATERIAL*

Multilayer Density Analysis of Cellulose Thin Films

Carina Sampl^1,2^, Katrin Niegelhell^1,2^, David Reishofer^1^, Roland Resel^3^, Stefan Spirk^1,2.*^, Ulrich Hirn^1,2^

^1^Institute for Paper, Pulp and Fibre Technology, Graz University of Technology, Graz, Austria

^2^Institute of Solid State Physics, Graz University of Technology, Graz, Austria

^3^CD-Laboratory for Fibre Swelling and Paper Performance, Graz University of Technology, Graz, Austria

Members of NAWI Graz and the European Polysaccharide Network of Excellence (EPNOE)

# LIST OF ABBREVATIONS.

| **GENERAL** |  |  |  |
| --- | --- | --- | --- |
| TMSC | … | Trimethylsilylcellulose |  |
| TMCS | … | Chlortrimethylsilan |  |
| n | … | refractive index |  |
| d | … | thickness | [nm] |
| a | … | cellulose content | [%] |
| dn/dλ | … | chromatic dispersion | [µm^-1^] |
| ρ | … | density | [g∙cm^-3^] |
| k_SP_ | … | surface plasmon wave vector |  |
| R_q_ | … | root-mean-square roughness | [nm] |
| RL | … | roughness layer |  |
| RLT | … | roughness layer thickness | [nm] |
| BL | … | bulk layer |  |
| BLT | … | bulk layer thickness | [nm] |
| mf | … | material* fraction | [%] |
|  |  |  |  |
| **SPR – AFM** | | |  |
| n_b_ | … | refractive index of bottom layer determined by MP-SPR |  |
| n_t_ | … | refractive index of top layer determined by MP-SPR |  |
| n^SPR^ | … | refractive index of whole cellulose thin film determined by MP-SPR |  |
| n_film_ | … | refractive index of whole cellulose thin film calculated (MP-SPR) |  |
| n_cellulose_ | … | refractive index of cellulose (n_cellulose_ = 1.467 (670nm),  1.4644(785nm)) |  |
| n_medium_ | … | refractive index ambient medium° |  |
| ρ_m_^b^ | … | density material* of bottom layer determined by MP-SPR | [g∙cm^-3^] |
| ρ_BL_ | … | density of bulk layer determined by AFM | [g∙cm^-3^] |
| ρ_m_^t^ | … | density material* of top layer determined by MP-SPR | [g∙cm^-3^] |
| ρ^SPR^ | … | density of whole cellulose thin film determined by MP-SPR | [g∙cm^-3^] |
| ρ_film_ | … | density of cellulose thin film calculated (MP-SPR) | [g∙cm^-3^] |
| ρ_cellulose_ | … | density of cellulose (ρ_cellulose_ = 1.5 g∙cm^-3^) | [g∙cm^-3^] |
| ρ_medium_ | … | density of ambient medium° | [g∙cm^-3^] |
| ρ_RL_^SPR^ | … | density of roughness layer determined by MP-SPR | [g∙cm^-3^] |
| ρ_RL_^AFM^ | … | density of roughness layer determined by AFM | [g∙cm^-3^] |
|  |  |  |  |
| d^SPR^ | … | thickness of whole cellulose thin film determined by MP-SPR | [nm] |
| d_b_ | … | thickness of bottom layer determined by MP-SPR | [nm] |
| d_t_ | … | thickness of top layer determined by MP-SPR | [nm] |
|  |  |  |  |
| a_b_^SPR^ | … | cellulose content of bottom layer determined by MP-SPR | [%] |
| a_t_^SPR^ | … | cellulose content of top layer determined by MP-SPR | [%] |
|  |  |  |  |
| dn/dλ_film_ | … | chromatic dispersion of cellulose thin film calculated | [µm^-1^] |
| dn/dλ_cellulose_ | … | chromatic dispersion of cellulose | [µm^-1^] |
| dn/dλ_medium_ | … | chromatic dispersion of ambient medium° | [µm^-1^] |
|  |  |  |  |
| RLT^SPR^ | … | roughness layer thickness calculated (MP-SPR) | [nm] |
| RLT^AFM^ | … | roughness layer thickness determined by AFM | [nm] |
| mf^SPR^ | … | material* fraction of roughness layer determined by MP-SPR | [%] |
| mf^AFM^ | … | material* fraction of roughness layer determined by AFM | [%] |

* material = cellulose + air

° ambient medium: *e.g*. air, H_2_O, etc.

BLT = d_b_

ρ_BL_ = ρ_m_^b^

# AFM Tomography evaluation.

**
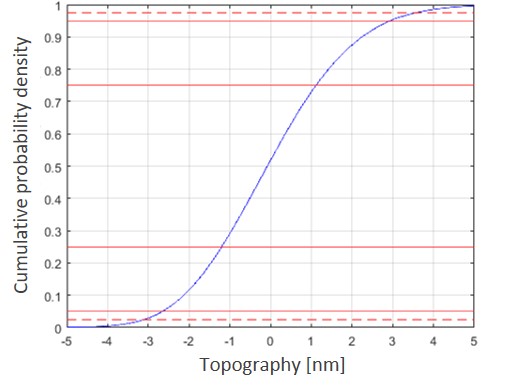
**

**FIGURE S1.** AFM topography evaluation (cumulative probability density vs. topography [nm]) for cellulose thin films spin coated from CHCl_3_. Calculated roughness layer thickness *RLT^AFM^* = 6.4 nm and material fraction *mf^AFM^* = 47.9 %.

# MP-SPR spectroscopy evaluation.

**
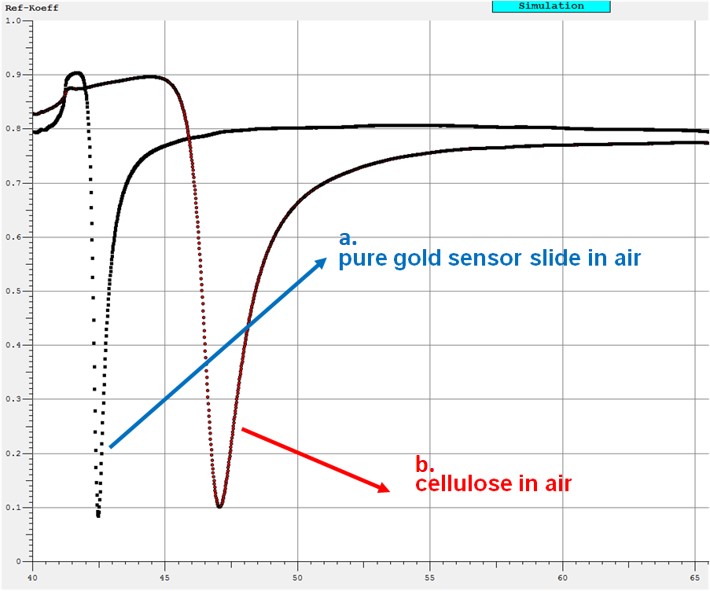
**

**FIGURE S2.** Pure gold sensor slide (background) (a) and cellulose thin film spin coated from CHCl_3_ measured in air (b).

# Calculation of film/layer density.

Densities of the layers in the film were calculated from the cellulose content *a* (of the corresponding layer) obtained from MP-SPR spectroscopy measurements according to the following equation.

$\rho_{film/layer}= a\cdot\rho_{cellulose}+\left( 1-a \right)\cdot\rho_{air}$ (13)

$$\rho_{cellulose}=1.5 g\cdot{cm}^{-3}$$

$$\rho_{air}=0.00118 g\cdot{cm}^{-3}$$

**TABLE S1.** MP-SPR simulation values (multi-layer analysis) at the example of a cellulose thin film spin coated from CHCl_3_ – according to **Scheme 1**, adopting equations 7-8 (see manuscript) ^a^.

|  | **λ1 (670 nm)** | | | **λ2 (785 nm)** | | |
| --- | --- | --- | --- | --- | --- | --- |
|  | **d [nm]** | **RI - n** | **kappa** | **d [nm]** | **RI - n** | **kappa** |
| **Simulation of SPR-curve in optical fitting software WINSPALL 3.01** | | | | | | |
| **Cr**^a^ | 4.80 | 1.300 | 11.049 | 4.98 | 1.315 | 13.384 |
| **Au**^a^ | 49.83 | 0.091 | 2.264 | 49.97 | 0.085 | 2.527 |
| **CELLULOSE** | 35.87 | 1.417 |  | 36.17 | 1.421 |  |
| **STEP 1: evaluation (2-λ method) of WHOLE cellulose layer** | | | | | | |
|  | d^SPR^ | n^SPR^ |  | d^SPR^ | n^SPR^ |  |
| **CELLULOSE** | **36.91** | **1.405** |  | **36.91** | **1.403** |  |
| **STEP 2: splitting of CELLULOSE film by implementing RLT^AFM^/2 for TOP layer thickness** | | | | | | |
|  | d_b_^0^ | n^SPR^ |  | d_b_^0^ | n^SPR^ |  |
| **BOTTOM** | **33.71** | 1.405 |  | **33.71** | 1.403 |  |
|  | d_t_^0^ | n^SPR^ |  | d_t_^0^ | n^SPR^ |  |
| **TOP** | **3.20** | 1.405 |  | **3.20** | 1.403 |  |
| **STEP 3: iteration of n^SPR^ in fitting program to obtain n_b/t_^0^** | | | | | | |
|  | d_b_^0^ | n_b_^0^ |  | d_b_^0^ | n_b_^0^ |  |
| **BOTTOM** | 33.71 | **1.422** |  | 33.71 | **1.418** |  |
|  | d_t_^0^ | n_t_^0^ |  | d_t_^0^ | n_t_^0^ |  |
| TOP | 3.20 | **1.214** |  | 3.20 | **1.239** |  |
| **STEP 4: evaluation (2-λ method) of BOTTOM layer, while TOP is constant** | | | | | | |
|  | d_b_^1^ | n_b_^1^ |  | d_b_^1^ | n_b_^1^ |  |
| **BOTTOM** | **33.11** | **1.429** |  | **33.11** | **1.427** |  |
|  | d_t_^0^ | n_t_^0^ |  | d_t_^0^ | n_t_^0^ |  |
| TOP | 3.20 | 1.214 |  | 3.20 | 1.239 |  |
| **STEP 5: implemention of RLT^AFM^ as d_t_^1^ - thickness of TOP layer** | | | | | | |
|  | d_b_^1^ | n_b_^1^ |  | d_b_^1^ | n_b_^1^ |  |
| BOTTOM | 33.11 | 1.429 |  | 33.11 | 1.427 |  |
|  | RLT^AFM^ (d_t_^1^) | n_t_^0^ |  | RLT^AFM^ (d_t_^1^) | n_t_^0^ |  |
| **TOP** | **6.40** | 1.214 |  | **6.40** | 1.239 |  |
| **STEP 6: iteration of n_t_^0^ in fitting program to obtain n_t_^1^** | | | | | | |
|  | d_b_^1^ | n_b_^1^ |  | d_b_^1^ | n_b_^1^ |  |
| BOTTOM | 33.11 | 1.429 |  | 33.11 | 1.427 |  |
|  | d_t_^1^ | n_t_^1^ |  | d_t_^1^ | n_t_^1^ |  |
| **TOP** | 6.400 | **1.192** |  | 6.40 | **1.168** |  |
| **STEP 7: evaluation (2-λ method) of TOP layer, while BOTTOM is constant** | | | | | | |
|  | d_b_^1^ | n_b_^1^ |  | d_b_^1^ | n_b_^1^ |  |
| BOTTOM | 33.11 | 1.429 |  | 33.11 | 1.427 |  |
|  | d_t_^2^ | n_t_^2^ |  | d_t_^2^ | n_t_^2^ |  |
| **TOP** | **3.31** | **1.272** |  | **3.31** | **1.270** |  |
| **STEP 8: re-evaluation (2-λ method) of BOTTOM layer** | | | | | | |
|  | d_b_^2^ | n_b_^2^ |  | d_b_^2^ | n_b_^2^ |  |
| **BOTTOM** | **33.22** | **1.428** |  | **33.22** | **1.425** |  |
|  | d_t_^2^ | n_t_^2^ |  | d_t_^2^ | n_t_^2^ |  |
| TOP | 3.31 | 1.272 |  | 3.31 | 1.270 |  |

^a^values for Cr and Au remain constant during the analysis.

**TABLE S2.** Multilayer analysis (*d_t_*, *ρ_m_^t^*, *d_b_*, *ρ_m_^b^*) results (equations 7-8, 13) – whole film, top layer, bottom layer - for the 3 cellulose thin films obtained by combination of MP-SPR (*d^SPR^*, *ρ^SPR^*) and AFM measurements.

|  | **film 1** | **film 2** | **film 3** | **average** | **sd** |
| --- | --- | --- | --- | --- | --- |
| **d^SPR^ [nm]** | 36.9 | 37.2 | 36.4 | 36.8 | 0.4 |
| **ρ^SPR^ [g∙cm^-3^]** | 1.34 | 1.33 | 1.35 | 1.34 | 0.01 |
| **cellulose content [%]** | 89.0 | 88.9 | 89.9 | 89.3 | 0.6 |
| **d_b_[nm]** | 33.2 | 34.3 | 33.6 | 33.7 | 0.6 |
| **ρ_m_^b^ [g∙cm^-3^]** | 1.42 | 1.38 | 1.41 | 1.40 | 0.02 |
| **cellulose content [%]** | 94.4 | 92.2 | 93.9 | 93.5 | 1.2 |
| **d_t_  [nm]** | 3.3 | 2.4 | 3.0 | 2.9 | 0.5 |
| **ρ_m_^t^ [g∙cm^-3^]** | 0.88 | 0.65 | 1.17 | 0.90 | 0.26 |
| **cellulose content [%]** | 58.3 | 43.1 | 77.8 | 59.7 | 17.4 |

**TABLE S3.** Results (*d_t_*, *ρ_m_^t^*, *d_b_*, *ρ_m_^b^*) of multilayer analysis (equations 7-8, 13) – whole film, top layer, bottom layer – calculated from multiple evaluations of each cellulose thin film measured by SPR (*d^SPR^*, *ρ^SPR^*) and AFM.

|  | **film 1** | | **film 2** | | **film 3** | | **overall** | |
| --- | --- | --- | --- | --- | --- | --- | --- | --- |
|  | **average** | **sd** | **average** | **sd** | **average** | **sd** | **average** | **sd** |
| **d^SPR^ [nm]** | 36.0 | 0.7 | 37.0 | 0.3 | 36.2 | 0.6 | 36.4 | 0.5 |
| **ρ^SPR^ [g∙cm^-3^]** | 1.36 | 0.15 | 1.30 | 0.07 | 1.37 | 0.07 | 1.34 | 0.09 |
| **cellulose content [%]** | 90.0 | 0.1 | 86.7 | 0.1 | 89.8 | 0.0 | 88.8 | 0.1 |
| **d_b_[nm]** | 32.9 | 0.5 | 34.1 | 0.7 | 34.1 | 0.9 | 33.7 | 0.7 |
| **ρ_m_^b^ [g∙cm^-3^]** | 1.42 | 0.04 | 1.36 | 0.02 | 1.38 | 0.05 | 1.39 | 0.04 |
| **cellulose content [%]** | 94.8 | 0.1 | 88.8 | 0.1 | 91.7 | 0.1 | 91.8 | 0.1 |
| **d_t_  [nm]** | 3.3 | 0.3 | 2.4 | 0.0 | 2.6 | 0.2 | 2.8 | 0.2 |
| **ρ_m_^t^ [g∙cm^-3^]** | 0.73 | 0.15 | 0.65 | 0.00 | 0.89 | 0.17 | 0.76 | 0.10 |
| **cellulose content [%]** | 48.3 | 0.1 | 43.1 | 0.0 | 65.3 | 0.2 | 52.2 | 0.1 |

**TABLE S4**. XRR results of a cellulose thin film (three-layer fit). Layer 1 corresponds to the small fraction of the thin film, which is directly attached to the substrates surface, followed by layer 2, which correlates to the multilayer analysis’ bottom layer. On top of these, the top layer (layer 3) or surface fraction of the thin film can be found, this layer interacts with the surrounding medium (*e.g.* air, liquids).

|  | **Layer 1** | | | **Layer 2 (= bottom layer)** | | | **Layer 3 (= top layer)** | | | **Total Thickness** | |
| --- | --- | --- | --- | --- | --- | --- | --- | --- | --- | --- | --- |
| **% RH** | **nm** | | **g∙cm^-3^** | **nm** | | **g∙cm^-3^** | **nm** | | **g∙cm^-3^** | **nm** | **%** |
|  | **Thickness** | **Roughness** | **Density** | **Thickness** | **Roughness** | **Density** | **Thickness** | **Roughness** | **Density** | **Thickness** | **Thickness** |
| **0** | 0.6 | 0.2 | 0.80 | 40.4 | 2.7 | 1.47 | 4.2 | 1.7 | 1.10 | 45.2 | 0.0 |
| **25** | 0.6 | 0.2 | 0.76 | 41.9 | 2.3 | 1.45 | 4.3 | 1.7 | 1.08 | 46.8 | 3.7 |
| **50** | 0.7 | 0.2 | 0.83 | 43.9 | 1.8 | 1.39 | 4.2 | 1.7 | 1.05 | 48.7 | 7.9 |
| **70** | 0.6 | 0.2 | 0.86 | 46.4 | 2.2 | 1.40 | 4.7 | 1.8 | 1.01 | 51.7 | 14.4 |
